# Real-time monitoring of vitamin C levels in trauma patients by electron-spin resonance spectrometry

**DOI:** 10.1186/s12873-023-00857-z

**Published:** 2023-08-05

**Authors:** Ryuichi Takenaka, Shigekiyo Matsumoto, Shinichi Nureki, Shinsuke Wada, Yoshimasa Oyama, Teruo Sakamoto, Takaaki Kitano, Osamu Shigemitsu

**Affiliations:** 1 Department of Emergency Medicine, Oita University, 1-1 Idaigaoka, Hasama-Machi, Yufu, Oita 879-5593 Japan; 2 Department of Anesthesiology and Intensive Care Medicine, Oita University, 1-1 Idaigaoka, Hasama-Machi, Yufu, Oita 879-5593 Japan; 3 Department of Respiratory Medicine and Infectious Disease, Oita University, 1-1 Idaigaoka, Hasama-Machi, Yufu, Oita 879-5593 Japan; 4 Advanced Trauma, Emergency and Critical Care Center, Oita University Hospital, 1-1 Idaigaoka, Hasama-Machi, Yufu, Oita 879-5593 Japan

**Keywords:** Trauma, Critical illness, Vitamin C, Electron-spin resonance spectrometry, Emergency

## Abstract

**Background:**

In critically ill patients, healthy vitamin C levels are important to avoid an imbalance in reactive oxygen species. To achieve this, oxidative stress levels in emergency patients need to be accurately measured in real-time. However, normally, reactive oxygen/nitrogen species are short-lived, rendering measurement difficult; moreover, measurement of relatively stable antioxidants and other oxidative stress markers in real-time is challenging. Therefore, we used electron-spin resonance spectrometry (ESR) to assess vitamin C levels, clarify their relationship with patients’ severity, and establish more effective vitamin C therapy in critically ill patients.

**Methods:**

We studied 103 severely ill emergency patients and 15 healthy volunteers. Vitamin C radical (VCR/dimethyl sulfoxide [DMSO]) values were analyzed in arterial blood samples by ESR at admission and once daily thereafter during the acute recovery phase. Severity scores were calculated. The relationship between these scores and VCR/DMSO values and chronological changes in VCR/DMSO values were analyzed.

**Results:**

Serum VCR/DMSO values were significantly lower in critically ill patients than in healthy volunteers (0.264 ± 0.014 vs. 0.935 ± 0.052, *p* < 0.05), particularly in the severe trauma group and the cardiopulmonary arrest/post-cardiac arrest syndrome group. VCR/DMSO values and various severity scores did not correlate at admission; however, they correlated with SOFA scores from days 2–6. VCR/DMSO values remained low from the first measurement day through Day 6 of illness.

**Conclusions:**

Vitamin C levels were low at admission, remained low with conventional nutritional support, and did not correlate with the initial patient’s severity; however, they correlated with patients’ severity after admission. Some patients had normal vitamin C levels. Therefore, vitamin C levels should be measured in real-time and supplemented if they are below normal levels.

**Trial registration:**

Retrospectively registered.

## Background

As oxidative stress in patients with conditions such as polytrauma and severe sepsis has a negative effect on prognoses [1], acute control of oxidative stress is crucial. However, patients brought to critical care centers include not only those with pre-existing conditions that cause chronic oxidative stress, such as diabetes [2], hypertension [3], dialysis [4], and aging [5], but also include many previously healthy patients with no underlying conditions that increase oxidative stress. Furthermore, some patients are brought in directly after the injury, while others are brought in after a considerable time since the injury. Consequently, the amount of oxidative stress at admission to a critical care center is expected to vary widely among individual cases, even if the level of invasiveness or trauma is similar.

Oxidative stress is caused by a breakdown in the balance between reactive oxygen/nitrogen species and antioxidants: an excess of reactive oxygen/nitrogen species or a depletion of antioxidants [6]. Several previous studies have attempted to treat emergency patients by administering high doses of antioxidants to control oxidative stress by suppressing reactive oxygen/nitrogen species; while some reported this to be effective [7, 8], others found it ineffective [9, 10]. Thus, the efficacy of this treatment remains uncertain.

We considered that administering uniformly high doses of antioxidants to emergency patients without accurately assessing each patient’s level of oxidative stress may be problematic. Reactive oxygen/nitrogen species play an important role in immune function and protect against infection and are vital in intracellular signaling and cellular processes such as differentiation and apoptosis [11–13]. Therefore, a simplistic assumption that all reactive oxygen/nitrogen species should be eliminated could be dangerous. Consequently, reactive oxygen/nitrogen species should not be overly suppressed, but oxidative stress should be controlled while maintaining a balance between reactive oxygen/nitrogen species and antioxidants. To achieve this, oxidative stress levels in emergency patients need to be accurately measured in realtime. However, normally, reactive oxygen/nitrogen species are short-lived, rendering measurement difficult, and even relatively stable antioxidants and other oxidative stress markers are impossible to measure in real-time, hindering the accurate evaluation of oxidative stress levels in emergency patients at critical care centers [14].

To address this, we focused on vitamin C [15], the antioxidant that responds most rapidly to oxidative stress and changes most markedly among the relatively stable antioxidants in vivo. Usually, serum vitamin C levels are measured through high-performance liquid chromatography, which is time-consuming and not applicable in a clinical setting. We developed a method for measuring vitamin C in real-time using an electron-spin resonance (ESR) spectrometer and applied this method in previous clinical research studies on acute respiratory disease syndrome (ARDS) [16], embryology [17], and sepsis [18]. In the present study, an ESR apparatus in an operating room adjacent to the critical care center was used to measure the serum vitamin C levels of emergency patients brought to the center in real-time. Changes in serum vitamin C levels in patients who had not been supplemented with vitamin C or other antioxidants were measured to investigate the necessity of vitamin C supplementation therapy in emergency patients.

## Methods

### Study population

This study was conducted on an opt-out basis. The research protocol adhered to the tenets of the Declaration of Helsinki and was conducted with approval from the relevant ethics committee. The opt-out option was posted on the notice board in the waiting room of the institution and on the home page of the institution's Advanced Trauma, Emergency and Critical Care Center. Written consent was obtained from participating healthy volunteers.

Blood samples were collected from emergency patients brought to the institution's Advanced Trauma, Emergency and Critical Care Center between April 1, 2016, and March 31, 2020. Of these patients, 103 provided blood samples for this study; among them, 69 had severe traumatic injuries, 23 had cardiopulmonary arrest/post-cardiac arrest syndrome, and 11 had other conditions, which included sepsis, acute respiratory distress syndrome, stroke, gastrointestinal hemorrhage, carbon monoxide poisoning, and tetanus. Fifteen healthy volunteers were also sampled as a control group.

Patients who were brought to the center after being treated at another hospital, patients from whom no blood sample was taken, and patients whose samples did not have enough blood remaining after clinical tests were excluded from the study.

### Measurement of serum vitamin C radicals (VCR)/dimethyl sulfoxide (DMSO) values

The blood samples were collected from radial arteries or femoral arteries for treatment purposes directly after patients were admitted to the hospital. The blood was centrifuged at 700 × g and 4 °C for 15 min, and the serum was collected. Serum VCR were measured as reported in previous studies [16].

**Fig. 1:**
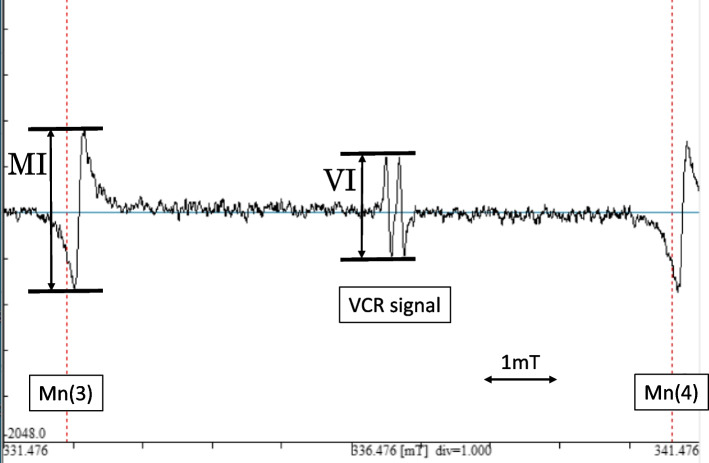
Electron-spin resonance (ESR) spectra of Vitamin C radicals (VCR) and manganese oxide (Mn)

The serum to be measured was divided into 50 µl aliquots, to which 100 µl DMSO (Sigma-Aldrich, St. Louis, MO, USA) was added. The mixtures were agitated for 10 s, and VCR values were measured by ESR (JES-FR30, JEOL Ltd, Tokyo, Japan) (Fig. 1). VCR signals that appeared between the third (Mn (3)) and fourth (Mn (4)) signals of the internal standard marker (manganese oxide Mn]) were measured. The strength of the Mn signals was labeled as MI, while the strength of the VCR signals was labeled as VI. The relative value of the VCR value divided by the Mn value (VI/MI) was recorded as the serum VCR/DMSO value.

If the VCR/DMSO value measurement at the collection time was not possible, the samples were stored in a dedicated freezer at -80℃. On the measurement day, the specimens were removed from the freezer and allowed to thaw slowly to room temperature. They were then centrifuged once again at 700 × *g* and 4℃ for 15 min. A 50-μl supernatant sample was extracted, and the VCR/DMSO value was measured according to the method detailed above. Previous research confirmed that there were no differences between newly drawn samples and values measured after 1, 2, or 3 months of storage (data not shown). Thus, it was considered that measuring stored samples for this study would not have an impact on the results.

### Severity scores

The Sequential Organ Failure Assessment (SOFA) score was calculated to indicate severity. This score reflects the severity of a patient’s organ damage [19, 20]. This score was used to investigate the relationship between vitamin C concentration and the patient’s status. For cases where the SOFA score was calculated from medical record data (*n* = 81), the relationship between the SOFA score at the time of blood sampling and the serum VCR/DMSO value was analyzed. When these data were lacking, patients were excluded from the analysis.

For traumatic injury patients, data from the medical records were used to calculate the Injury Severity Score (ISS) from the Abbreviated Injury Scale (AIS)-2005 2008 update [21, 22]. The AIS assigns codes to each individual traumatic injury. The codes are then integrated by anatomical location to generate the ISS, which indicates the severity of anatomical trauma to the whole body. This score was used to reflect the anatomical severity of the traumatic injury. Additionally, the Revised Trauma Score (RTS) was used as an index to calculate the physiological severity of the traumatic injury [23]. Age factors were added to the ISS and RTS to use the Trauma and Injury Severity Score (TRISS) method to calculate the probability of survival (Ps) [24]. In addition, we investigated the relationship of serum VCR/DMSO levels with the ISS, RTS, and Ps.

### Chronological changes in VCR/DMSO values

The changes in values over time were analyzed for cases in which the VCR/DMSO values could be measured at multiple time points. The day of admittance to the hospital was set as Day 1, and patients were evaluated until Day 6. As the study used an opt-out approach, the number of days for which patients were sampled differed by patient: Day 1 (day of admission), *n* = 103; Day 2, *n* = 32; Day 3, *n* = 29; Day 4, *n* = 20; Day 5, *n* = 16; and Day 6 *n* = 15. Serum VCR/DMSO levels on Day 1 were compared with those on each subsequent day.

### Statistical analysis

The results are presented as average ± standard error. A *t*-test was performed to compare age as a patient background factor between the healthy and patient groups. The sex ratio and smoking status were compared between these groups through a chi-squared test. The Mann‒Whitney U test was used to compare VCR/DMSO values among different groups. Changes in VCR/DMSO values over time were analyzed through analysis of variance and Dunnett’s multiple comparison test. The correlation between VCR/DMSO values and each severity score was analyzed through Spearman’s Rank Correlation test. P values of < 0.05 were considered statistically significant. Analyses were performed using the statistical analysis software GraphPad Prism version 9.3.1 for Windows (GraphPad Software, La Jolla, CA, USA).

## Results

### Participant background

The background characteristics of the participants are shown in Table 1. For the healthy group, the average age was 38.5 years, while for the emergency patient group, the average age was 63.1 years (*p* < 0.05). In the healthy group, 73.3% were men (11/15 cases), while in the emergency patient group, 75.7% were men (78/103 cases) (*p* = 0.20). The proportion of smokers was 26.7% (4/15 cases) in the healthy group and 29.6% (24/81 cases) in the emergency patient group (*p* = 0.82). The death rate as of Day 28 in the emergency patient group was 15.5% (16/103 cases).

**Table 1:** Baseline characteristics of the patients

	Healthy volunteers	Emergency patients
No. of individuals	*N* = 15	*N* = 103
		Traumatic injury: *n* = 69
		Post-cardiac arrest: *n* = 23
		Other: *n* = 13
		Sepsis: *n* = 3
		ARDS: 3
		Stroke: *n* = 2
		Carbon monoxide poisoning: *n* = 1
		Tetanus: *n* = 1
		Gastrointestinal hemorrhage: *n* = 1
Age (range), years	38.5 (23‒67)	63.1 (14‒91)
Male/female	11/4	78/25
Smoker/non-smoker/unclear	4/11/0	24/57/23
Survived/died	15/0	87/16

Ages are shown as average values (range: youngest‒oldest). The number of patients are reported for sex, smoking status, and 28-day survival rate

*Abbreviations*: *ADRS* Acute respiratory distress syndrome

### VCR/DMSO values at time of admission

**Fig. 2:**
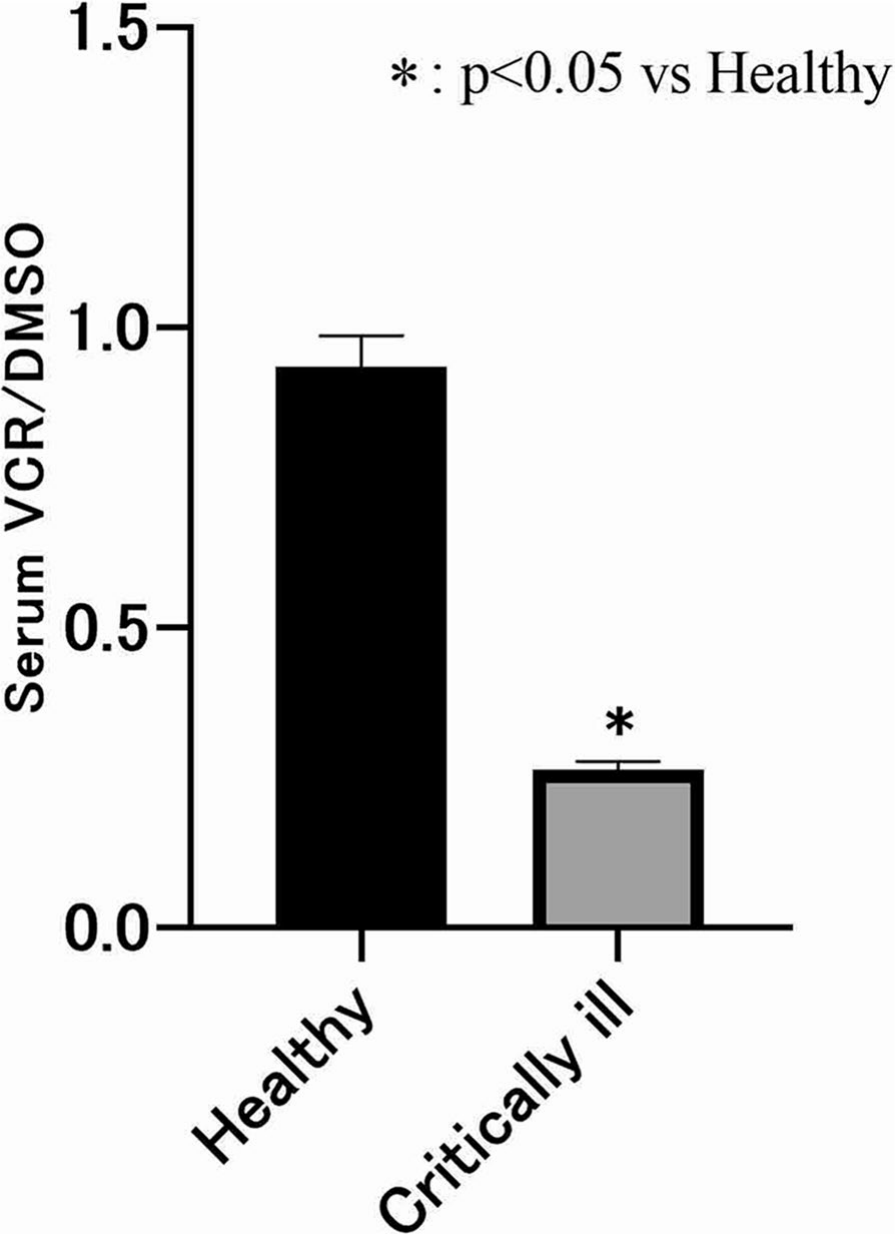
Comparison of VCR/DMSO levels in critically ill patients upon emergency room admission and healthy volunteers. Data are presented as the mean ± SEM. Analysis was performed using the Mann‒Whitney U test. Significantly lower levels were observed in critical patients compared to healthy volunteers (*p* < 0.05)

All serum VCR/DMSO values for emergency patients and healthy volunteers are shown in Fig. 2. Overall, the mean serum VCR/DMSO value for the emergency patient group was 0.264 ± 0.014, which was significantly lower than the value for the healthy group (0.935 ± 0.052; *p* < 0.01).

**Fig. 3:**
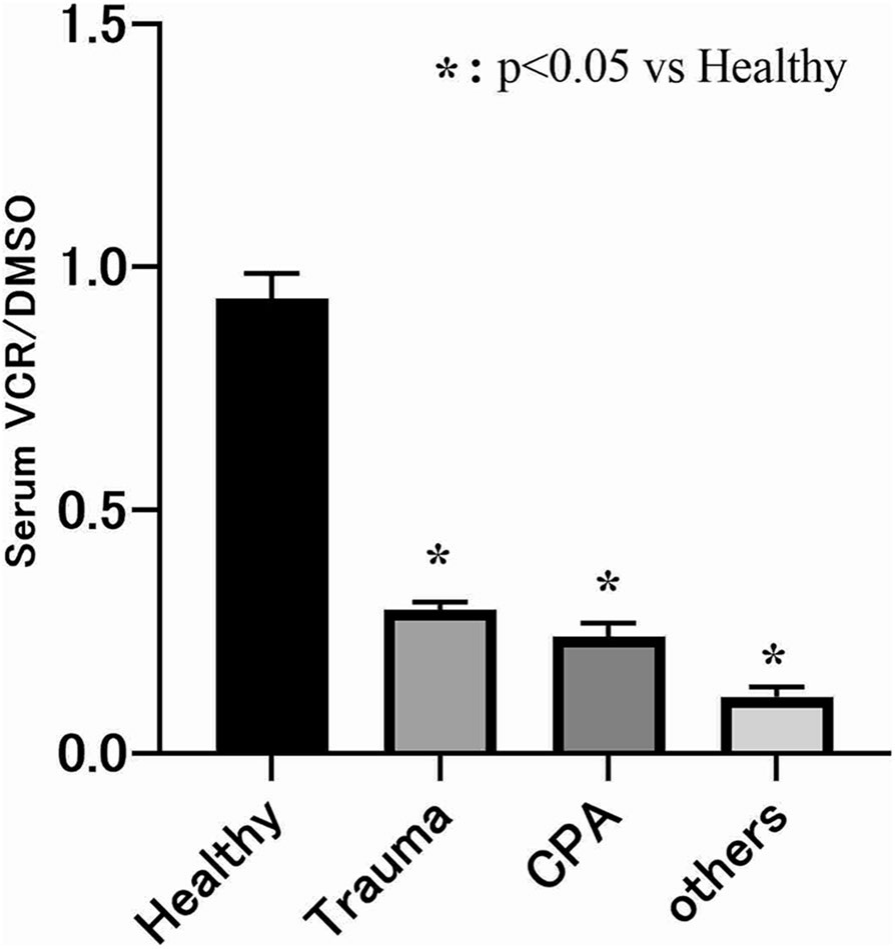
Serum VCR/DMSO levels in critically ill patients upon emergency room admission. Data are presented as the mean ± SEM. Group comparisons were conducted using the Mann‒Whitney U test. Abbreviations: Healthy—healthy volunteers, Trauma—trauma patients, CPA—cardiopulmonary arrest and post-cardiac arrest syndrome patients, Others—patients with other conditions

The serum VCR/DMSO levels for each critically ill group are shown in Fig. 3. The mean values were 0.295 ± 0.016, 0.241 ± 0.027, and 0.116 ± 0.021 for the traumatic injury, cardiopulmonary arrest/post-cardiac arrest syndrome, and other groups, respectively. Values were significantly lower for each group than for the healthy group (p < 0.01). No significant difference was seen between the values for the traumatic injury group and those for the cardiopulmonary arrest/post-cardiac arrest syndrome group (*p* = 0.07).

### Chronological changes in VCR/DMSO values

**Fig. 4:**
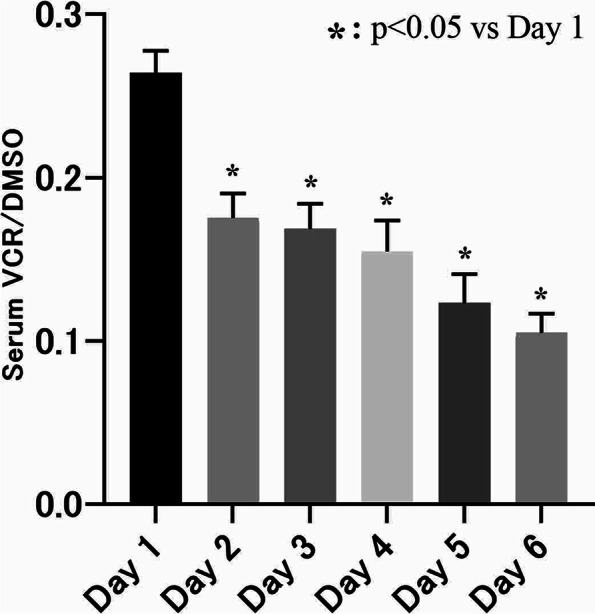
Serum VCR/DMSO levels in critically ill patients after emergency room admission. Data are presented as the mean ± SEM. Dunnett's multiple comparison test was used for analysis

Changes in VCR/DMSO values over time are shown in Fig. 4. Analysis of variance showed significant changes in the values over time (*p* < 0.05). Dunnett’s multiple comparison test showed that values were significantly lower on all subsequent days than on the Day 1 (*p* < 0.05). This finding suggests that vitamin C is depleted in emergency patients receiving only normal nutritional management.

### Relationship between VCR/DMSO values and severity scores

For the cases where the SOFA score could be calculated from medical records (*n* = 81), the correlation between the SOFA score and VCR/DMSO values was analyzed. No significant correlation was found between the SOFA score and VCR/DMSO values at admission (Table 2). In addition, for the traumatic injury patients (*n* = 69), the correlation of the VCR/DMSO values with the ISS score, RTS, and Ps calculated using the TRISS method was analyzed. No significant correlation was found between any of the severity scores and the VCR/DMSO values at admission (Tables 2 and 3).

**Table 2:** Correlation between VCR/DMSO values and SOFA score parameters

	Day 1	Day 2	Day 3	Day 4	Day 5	Day 6
r-value	-0.194	-0.630	-0.578	-0.589	-0.652	-0.205
*p*-value	0.083	0.001	0.001	0.008	0.008	0.478

Data were analyzed using Spearman’s rank correlation. No significant correlation at admission. Significant negative correlation from Days 2–5

*Abbreviations*: *SOFA* Sequential Organ Failure Assessment score

**Table 3:** Correlation between VCR/DMSO values and traumatic score parameters

		Day 1	Day 2	Day 3	Day 4	Day 5	Day 6
ISS	r-value	-0.041	-0.392	-0.524	-0.387	-0.400	-0.295
	*p*-value	0.743	0.024	0.010	0.213	0.291	0.375
RTS	r-value	0.111	0.170	0.313	0.473	0.414	0.255
	*p*-value	0.368	0.346	0.146	0.123	0.333	0.445
Ps	r-value	0.126	0.342	0.327	0.235	0.500	0.310
	*p*-value	0.308	0.052	0.128	0.460	0.178	0.350

Data were analyzed using Spearman’s rank correlation. No significant correlations were seen for any score at the time of admission. Significant negative correlations between ISS score parameters and VCR/DMSO values from Days 2–3

*Abbreviations*: *ISS* Injury Severity Score, *RTS* Revised Trauma Score, *Ps* Probability of survival

A time-series analysis showed a significant negative correlation between the SOFA score and VCR/DMSO values from Days 2–5 (Table 2) and a significant positive correlation between the ISS score and VCR/DMSO values from Days 2–3 (Table 3). Furthermore, there was no significant correlation between VCR/DMSO values and RTS and between VCR/DMSO values and Ps (Table 3).

## Discussion

In severely ill patients with systemic inflammatory responses, excessive amounts of ROS released from activated neutrophils cause direct damage to the vascular endothelium, giving rise to microangiopathy [25, 26]. In addition, ROS activate NF-κB, promoting indirect inflammatory reactions [1]. Although vitamin C is the first line of defense against oxidative stress by eliminating ROS, humans cannot synthesize vitamin C, which is easily depleted during a sustained systemic inflammatory response. Accordingly, if vitamin C is not immediately supplemented, there is a possibility that the inflammatory response will spread through the entire body, and vascular endothelial damage will progress to cause multiple organ failure [27]. Furthermore, in the hypothalamus‒pituitary‒adrenocortical system, vitamin C is an important cofactor in synthesizing catecholamines [28, 29]. Therefore, when vitamin C is depleted, catecholamine levels decrease, possibly causing the patient to go into shock. Because of the above factors, it is believed that immediate administration of vitamin C to emergency patients during the acute phase can suppress the inflammatory response and the damage to the vascular endothelium, as well as enable swift recovery from shock and prevent multiple organ failure.

However, as ROS also play an important role in biological defenses and intracellular signaling [11], excessive levels of antioxidants can cause problems [30, 31]. For this reason, we considered that, when administering vitamin C to patients in critical condition, a balance between oxides and antioxidants should be maintained. That is, vitamin C should be administered according to real-time monitoring, and vitamin C levels should always be maintained within normal parameters.

While it is possible to measure vitamin C levels in real-time at the time of admittance by determining VCR/DMSO, it can also be assumed that the lower the vitamin C levels, the more extreme the oxidative stress, thus enabling the evaluation of oxidative stress in real-time. A previous study found that the VCR/DMSO value positively correlates with the vitamin C concentration measured by high-performance liquid chromatography and that the vitamin C concentration can be estimated from the VCR/DMSO value [16]. In this study, we determined the vitamin C levels of emergency patients in severe conditions at the time of hospital admission and demonstrated that they had significantly lower vitamin C levels than did healthy volunteers (Fig. 2). However, some of the emergency patients had normal vitamin C levels. In fact, vitamin C levels at the time of admission were not dependent on the severity of a patient’s condition, as shown in Table 2, which demonstrates the lack of correlation between vitamin C levels and various severity scores. These results could be interpreted as showing that other factors, such as emergency patients’ pre-existing conditions and lifestyle, as well as the conditions under which patients were transported to the hospital and the time between trauma and hospital admission, have a major impact on oxidative stress.

Many previous reports have shown significant depletion of vitamin C caused by oxidative stress under various conditions, such as sepsis, ARDS, and traumatic injury [32, 33]. The present study also showed decreased levels of vitamin C and excessive oxidative stress in patients with severe traumatic injury, cardiopulmonary arrest/post-cardiac arrest syndrome, and various other emergency conditions (Fig. 3). Figure 4 shows the change in vitamin C levels over time following admission to the hospital, but vitamin C levels remained low through Day 6 of the illness. The patients recorded in this study were provided with conventional nutritional support following admission, and they were dosed with less than 100 mg of vitamin C per day. This suggests that, under conventional nutrition management, vitamin C levels decreased over time and became depleted. If vitamin C levels remain depleted, vascular endothelial damage and organ damage can progress, and the patient’s recovery from shock may be delayed. The negative correlation between VCR/DMSO and SOFA scores over time in this study (Table 2) suggests that vitamin C deficiency might contribute to organ damage. Thus, vitamin C should be administered immediately. However, previous reports on the effectiveness of vitamin C in treating severely ill patients have yielded contradictory results, providing no clear evidence for its effectiveness. Scholz et al. reported that, in patients with sepsis, the patient group who were given vitamin C for 3‒4 days showed an improved prognosis over those who were given vitamin C for 1‒2 days for 5 or more days [34]. This suggests that administering too little or too much vitamin C is ineffective and supports this study’s hypothesis that it is important to supplement vitamin C while maintaining a balance between oxides and antioxidants. Furthermore, it is known that the biokinetics of vitamin C varies across individuals [35].

Overall, during the treatment of emergency patients, such as those with trauma, with vitamin C therapy, maintaining normal levels of vitamin C at all times is more effective than administering high doses. Therefore, future research should gather data on the optimal dosage, timing, and relationship between the treatment period and prognosis of vitamin C therapy. The real-time vitamin C measurement technique demonstrated in this study will facilitate the conduct of future studies.

This study has several limitations. As this study was conducted on an opt-out basis using the remainder of specimens taken for treatment purposes, patients whose remaining samples did not contain enough blood for the present study’s analyses could not be included. In addition, clinical parameters used to assess trauma severity were taken from medical records, but in some cases, information on the patient’s condition upon admission to the hospital and/or data records were missing. For the measurement of changes in vitamin C levels over time, the timing of the blood samples taken for treatment purposes varied among patients, meaning that sample sizes for each day of illness varied greatly.

## Conclusions

In conclusion, levels of vitamin C in critically ill patients, as measured by ESR, had already decreased significantly when patients were admitted to the hospital and continued to decrease and become depleted over time when only conventional nutritional support was provided. Furthermore, the serial decrease in vitamin C may be related to the degree of subsequent organ damage. Therefore, it is possible that swift administration of vitamin C to patients with severe trauma during the acute period may suppress vascular endothelial damage and the inflammatory response and may also help patients to recover more quickly from the shock. In addition, while there was no correlation between vitamin C levels and severity scores at admission, this correlation may be masked by differing levels of oxidative stress in each patient due to pre-existing conditions and lifestyles prior to the trauma. In the future, to establish more effective vitamin C supplementation therapy, trauma patients should not simply be given large amounts of vitamin C; rather, serum vitamin C concentrations should be monitored in real-time in order to determine the appropriate dosage, timing, and administration period. Additionally, future studies should investigate how these factors affect prognosis.

## Data Availability

The datasets used and analysed during the current study available from the corresponding author upon reasonable request.

## Abbreviations

AIS: Abbreviated Injury Scale
ARDS: Acute respiratory distress syndrome
CPA: Cardiopulmonary arrest
DMSO: Dimethyl sulfoxide
ESR: Electron-spin resonance spectrometry
ISS: Injury Severity Score
Mn: Manganese oxide
Ps: Probability of survival
ROS: Reactive oxygen species
RTS: Revised Trauma Score
SOFA: Sequential Organ Failure Assessment score
TRISS: Trauma and Injury Severity Score
VCR: Vitamin C radical
